# Effects of Trauma in Adulthood and Adolescence on Fear Extinction and Extinction Retention: Advancing Animal Models of Posttraumatic Stress Disorder

**DOI:** 10.3389/fnbeh.2018.00247

**Published:** 2018-10-31

**Authors:** Chieh V. Chen, Lauren E. Chaby, Sahana Nazeer, Israel Liberzon

**Affiliations:** ^1^Ann Arbor Veterans Affairs, Ann Arbor, MI, United States; ^2^Department of Psychiatry, University of Michigan, Ann Arbor, MI, United States; ^3^Department of Neuroscience, Brown University, Providence, RI, United States

**Keywords:** adolescence, single prolonged stress, PTSD, predation stress, developmental stress

## Abstract

Evidence for and against adolescent vulnerability to posttraumatic stress disorder (PTSD) is mounting, but this evidence is largely qualitative, retrospective, or complicated by variation in prior stress exposure and trauma context. Here, we examine the effects of development on trauma vulnerability using adult post-natal (PN) day 61, early adolescent (PN23) and mid adolescence (PN34) rats and two types of trauma: an established animal model of PTSD, single prolonged stress (SPS), and a novel composite model—SPS predation (SPSp) version. We demonstrate that early and mid adolescent rats are capable of fear conditioning and fear extinction, as well as extinction retention. Our results also demonstrate that both types of trauma induced a deficit in the retention of fear extinction in adulthood, a hallmark of PTSD, but not after early or mid adolescence trauma, suggesting that adolescence might convey resilience to SPS and SPSp traumas. Across all three life stages, the effects of SPS exposure and a novel predation trauma model, SPSp, had similar effects on behavior suggesting that trauma type did not affect the likelihood of developing PTSD-like symptoms, and that SPSp is a predation-based trauma model worth exploring.

**Highlights**

- Posttraumatic stress disorder (PTSD) risk is mediated by trauma type and age at exposure.
- We compare an established and novel composite trauma model in adolescence and adulthood.
- Both models induced extinction retention deficits in adulthood, but not adolescence.
- Adolescents had intact extinction and extinction retention after trauma, suggesting resilience.

## Introduction

It is commonly asserted in the literature that children and adolescents are at higher risk for trauma related psychopathology (Pynoos et al., 1987; North et al., 1994); however, the empirical/mechanistic evidence for this assertion is sparse and often contradictory (Green et al., 1991; Shannon et al., 1994; Tottenham and Gabard-Durnam, 2017; reviewed in Chaby et al., 2017). Indeed, increased vulnerability to posttraumatic stress disorder (PTSD) following trauma in adolescence has been supported by some (Green et al., 1991) but not other studies (McFarlane, 1987; Lonigan et al., 1991). These differences stem partly from challenges inherent in the study of clinical populations, such as variation between participants in trauma type, timing, duration and prior stress history (van der Kolk, 1985; Davidson and Smith, 1990; Bokszczanin, 2007; reviewed in Schwarz and Perry, 1994). While some studies examined the relationship between trauma type and PTSD symptom profile (Kelley et al., 2009; Price et al., 2013), the categorization of trauma type is hard as it depends on individual experiences, culture and individual history, that can also interact with the age at exposure (McCloskey and Walker, 2000; Avital and Richter-Levin, 2005; Ricon et al., 2012). Animal studies that systematically evaluate the effect of various trauma types across ontogeny can help to overcome some of these challenges by elucidating aspects of the traumatic response that are common across traumas.

With respect to the risk conveyed by the developmental stage itself, adolescents may have differential sensitivity to trauma because of states of maturation of brain tissues, developmental plasticity, immature capacity for emotion regulation, and age-specific differential cognitive coping skills (Spear, 2000; Crews et al., 2007; Semple et al., 2013). One of the ways developmental processes can alter vulnerability to post traumatic psychopathology, is via altered fear associated learning and extinction, which has been shown to be affected in both PTSD patients and animal models of PTSD in adulthood (Liberzon et al., 1997, 1999). In fact, it has been reported that contextually learned fear expression is temporarily suppressed in adolescence, a phenomenon modulated by developmental changes in brain derived neurotrophic factor (BDNF; Pattwell et al., 2011; Dincheva et al., 2014). In addition, trauma may also have differential effects across ontogeny on extinction retention, a capacity that is deficient in PTSD patients. Extinction retention has been linked to activation of the ventromedial prefrontal cortex (PFC) and the hippocampus in adults, areas that still undergo development in adolescence (Meaney et al., 1985; Sowell et al., 1999), and that show reduced activity in PTSD patients (Milad et al., 2007, 2009).

In the present study, we aimed to test the effects of trauma exposure in adult and adolescent rats using two different models of trauma. First, we used a well established trauma model, single prolonged stress (SPS), which has been used for nearly two decades to model PTSD-specific deficits in extinction retention (Knox et al., 2012; reviewed in Yamamoto et al., 2009). Second, we employed a novel predation model that used similar to SPS temporal characteristics. Predator models have been popular due to their face validity, and they had been shown to induce both overlapping and distinct symptoms of PTSD compared with SPS (Daskalakis et al., 2013; Deslauriers et al., 2017). For example, both predation models and SPS can induce heightened glucocorticoid receptor (GR) receptor levels, startle responsivity, anxiety-like behavior and pro-inflammatory cytokine levels (Khan and Liberzon, 2004; Yamamoto et al., 2009; Elharrar et al., 2013; Zoladz and Diamond, 2013; Lin et al., 2016; reviewed in Deslauriers et al., 2017). Yet, to the authors knowledge, SPS has been shown to result in PTSD-like sleep disturbances, extinction retention deficits and anhedonia (Perrine et al., 2016; Nedelcovych et al., 2015; Vanderheyden et al., 2015), while predation models have been shown to induce avoidance of trauma reminders and decreased dendritic length and spine density in the hippocampus (Cohen et al., 2011, 2014). Using SPS temporal characteristics (single prolonged exposure to multiple stressors, followed by a sensitization period) we created a novel model, SPS-predation (SPSp), to investigate potential effects of trauma type and developmental timing on fear learning and extinction retention. Given distinct psychopathological effects of trauma type at different developmental timepoints (McCloskey and Walker, 2000; Kelley et al., 2009), we predicted that the trauma models would have distinct effects on fear learning processes across three developmental stages, but that early and mid adolescents would have enhanced vulnerability to both trauma types because of immature neural networks for processing extinction retention. In addition, we further examined the validity of the SPSp model by testing their startle response, a response shown to be heightened in PTSD patients (Shalev et al., 2000) and in animals exposed to SPS (Khan and Liberzon, 2004).

## Materials and Methods

### Animals and Housing

Male Sprague-Dawley rats were obtained as adults (post-natal day [PN] 61, *n* = 48), mid adolescents (PN 34, *n* = 24) and early adolescents (PN 23, *n* = 24), from Charles River (Kingston, NY, USA). To describe developmental stages, we used nomenclature from Cicchetti (2016), which reflect sex organ maturation. The upper limit of the phase is defined by the emergence of sexual behavior. The second phase, mid adolescence (also called mid puberty), encompasses widespread maturation of the sex organs. The lower limit, termed early adolescence (also called early puberty), ends prior to the onset of most maturation processes in the sex organs (Spear, 2000; Cicchetti, 2016).

Following arrival, all rats were pair housed and allowed to acclimate for 4 days at the Veterinary Medical Unit of the Ann Arbor Veterans Affairs Medical Center. Rats were maintained on a 12:12 h light/dark cycle (lights on at 6 am), at 20–22°C, and 50% humidity. Rats were given *ad libitum* access to water and chow (25% protein, Laboratory Rodent Diet 5001, LabDiet). All procedures and protocols were approved by the Ann Arbor Veteran Affairs Institutional Animal Care and Use Committee (protocol #1312-004) and were in accordance with the National Institute of Health standard for the treatment of animals.

### Trauma Models

#### Single Prolonged Stress

In the SPS procedure, rats were exposed to three stressors in the first day (Liberzon et al., 1997) that target the hypothalamic-pituitary-adrenal axis via psychological, physiological and direct pituitary routs. Briefly, rats were restrained for 2 h, were exposed to a 20 min forced swim (68 × 56 × 45 cm container with water kept at 23–24°C) and, after a 15 min recuperation, were finally exposed to ether vapors in a desiccator until they lost consciousness and were fully anesthetized. Animals were then individually housed in clean cages and left undisturbed for 7 days, the time required for PTSD-like phenotype to develop (Liberzon et al., 1999; Knox et al., 2012). Control rats were also single housed starting on the day that SPS rats were exposed to trauma and left undisturbed for 7 days.

#### Single Prolonged Stress Predator Version

Similar to SPS, the SPSp, encompasses three stressors that are ecologically relevant to rodents. Animals were left in their home cages and exposed to a scent of a predator, fox urine (Tink’s Fox-P^®^), for 2 h. The scent was sprayed onto cotton balls and encased in a small ventilated plastic container, which was removed at the end of the 2 h (Chaby et al., 2015b). Following scent exposure, rats were exposed to a recording of predatory feline calls for 20 min in their home cages (Chaby et al., 2015a,b, 2016). Finally, animals were placed in an open arena (92 × 92 × 63 cm) for 5 min with a hawk shaped kite hanging overhead, which was lowered towards the rats to mimic approach (Chaby et al., 2015b). Rats were then transferred to clean cages, individually housed and left undisturbed for 7 days. Control rats were also single housed starting on the day that SPSp rats were exposed to trauma and left undisturbed for 7 days.

### Fear Conditioning, Fear Extinction, Extinction Recall

Rats first learned a fear association of a tone followed by a foot shock, which was extinguished the next day when animals were presented with the tone alone. On the last day, rats were again presented with tones alone to evaluate retention of extinction.

In detail, eight sound-attenuating boxes housed individual experimental chambers measuring 30 × 24 × 21 cm (MED Associates, St. Albans, VT, USA), which were connected to interfaces that controlled the experimental contingencies determined using MedPC software. The floor of each chamber was made of stainless steel rods measuring 4 mm in diameter and placed 1.5 cm apart from each other. The floor was connected to a shock source, which delivered a 1 s 1 mA shock unconditioned stimulus. The chambers were mounted with a speaker that delivered a 10 s 1 kHz 80 dB acoustic tone, which was used as the conditioned stimulus. Each chamber was also equipped with a 15 W light, and a fan that provided a 65 dB white noise.

Two distinct contexts with different auditory, visual, tactile and olfactory cues were used. Context A: lidded black boxes were used to transport the animals from their home cage to the testing chambers. Ammonium hydroxide (1%) was used as the scent cue and a room red light was used as the visual cue. The doors of the sound-attenuating boxes were left open, and the chamber lights and fans were left off. Context B: lidded white boxes with clean bedding were used to transport animals from their home cages to the testing chambers. Acetic acid (1%) was used as the scent cue, chamber lights as the visual cue, and chamber fans as the auditory cue. The doors of the sound-attenuating boxes were left closed.

For fear conditioning, context A was used. Baseline movement was recorded for 3 min before the animals were presented with five tones as the conditioned stimulus that co-terminated with a foot shock as unconditioned stimulus. For fear extinction and extinction recall, context B was used. Rats were left for 3 min in the chamber before being presented with 30 or 10 conditioned stimulus tones, respectively. Tones lasted 10 s and time between each conditioned stimulus was 1 min. For all three tests, animals were transported to the testing room and left to acclimate for 10 min in their transport boxes before testing. Transport boxes and testing chambers were cleaned with running tap water and dried between animals.

To minimize disturbance, animal behavior was recorded with an overhead camera on top of each chamber and the experimenter was not present in the room during testing. Percent freezing (total time between tones × 100/time freezing between tones) was analyzed using the Any-Maze Software (Stoelting, Co., Wood Dale, IL, USA). Freezing was defined as immobility, lasting longer than 1 s. Freezing behavior during a conditioned stimulus presentation and the following inter-trial interval was considered a single trial.

### Acoustic Startle

The startle chambers were 30 × 30 × 30 cm and housed a cylindrical tube 20 cm in length and 10 cm in diameter. The chamber was equipped with a 15 W light and speakers that delivered the acoustic bursts. Rats were allowed to acclimate to the acoustic chamber for 5 min. Thirty acoustic bursts (108 dB, 50 ms, 30 s intervals) were then delivered for 15 min and startle response (V_max_ = startle amplitude) was recorded and analyzed by an automated hardware/software package (San Diego Instruments, San Diego, CA, USA).

### Experiment 1: Trauma in Adulthood, Mid-puberty, or Early-Puberty and Susceptibility to Reduced Fear Extinction and Retention

To determine whether developmental stage affects fear associated learning after exposure to SPS, three different studies were run where rats at three different developmental stages were used: the first study used adult (PN61, *n* = 24), the second used mid adolescent (PN34, *n* = 24) and the third used early adolescent (PN23, *n* = 24) male rats. To evaluate whether effects on fear associated learning are SPS specific, we exposed animals in the above-mentioned developmental stages to the SPSp model. Thus, each study comprised of 24 rats of the same age group, and included the SPS, SPSp or control groups, resulting in eight rats per group.

**Figure 1:**
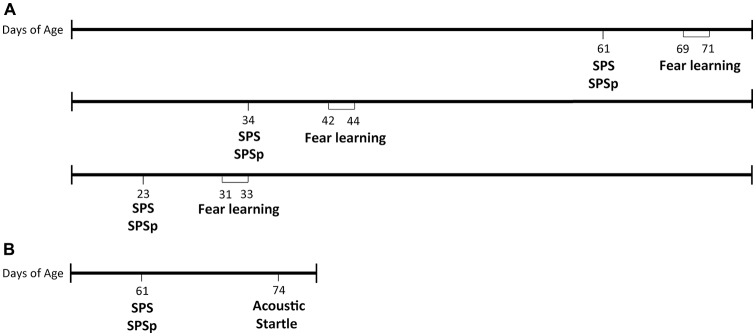
Timeline of procedures. **(A)** Experiment 1. **(B)** Experiment 2.

Four days after arrival, rats underwent SPS, SPSp, or, if assigned to an unstressed control group, were transferred to clean cages and singly housed for the remainder of the study. After 7 days, all rats underwent 3 days of testing: fear conditioning, extinction, and extinction recall (timeline in Figure 1A).

To eliminate the possibility that a group difference is falsely identified, we tested a separate cohort of adult animals (*n* = 8 per group) on the same paradigm mentioned above.

### Experiment 2: SPS and SPSp Modeling the PTSD Symptom of Acoustic Startle Response

Our laboratory previously reported that exposure to SPS enhances startle response (Khan and Liberzon, 2004), a phenotype also present in patients with PTSD. To determine whether effects of SPSp on startle response are similar to those resulting from SPS, we exposed 24 PN61 male rats to the SPS, SPSp or control procedures, and tested them on the acoustic startle test 13 days later (timeline in Figure 1B). Rats were single housed on the first day of SPS, SPSp or control procedures and remained so for the rest of the study.

### Statistical Analysis

Experiment 1: for all studies, fear learning data were analyzed with a repeated measures ANOVA with treatment (SPS, SPSp or control) as the between groups factor and trial (baseline, tone 1, tone 2, etc.) as a within groups factor. If a main effect of treatment was found, a *post hoc* least significant difference (LSD) analysis was used to compare marginal means. If an interaction effect was found, one-way ANOVAs were conducted to compare cell means. Rats that did not acquire conditioning (percent freezing remained below 30% for all fear conditioning trials; one rat in the adult Control group, two rats in the late adolescent control group, and one rat in the late adolescent SPS group), did not show recall of conditioning (percent freezing remained below 30% in the first five fear extinction trials; one rat in the late adolescent SPS group and three in the late adolescent SPSp group) were removed; and rats that showed freezing 2 standard deviations above or below the mean in half or more of the tones in one session were also removed (one rat in ER in the Control late adolescent group was removed). For fear extinction, percent freezing across three trials were averaged into a block, resulting in 10 blocks.

Experiment 2: startle amplitude across three acoustic bursts were averaged into a block, resulting in 10 acoustic burst blocks. A repeated measures ANOVA was conducted with treatment (SPS, SPSp or control) as the between subjects factor and acoustic burst (burst block 1, burst block 2… burst block 10) as a within subjects factor. If a main effect of treatment was found, a *post hoc* LSD analysis was used to compare marginal means. If an interaction effect was found, one-way ANOVAs were conducted to compare cell means.

## Results

### Experiment 1: Trauma in Early-Puberty, Mid-puberty, or Adulthood and Susceptibility to Reduced Fear Extinction and Retention

#### Adult Rats: (Figure 2A)

Fear conditioning: all animals showed increasing freezing through consecutive trials (*F* = 87.05, *p* < 0.001). There was no main effect of trauma exposure (*F* = 1.896, *p* = 0.176). An interaction effect was found (*F* = 3.938, *p* < 0.001) such that, at trial 3, control animals showed lower freezing compared to rats exposed to SPS (*p* = 0.007) and SPSp (*p* = 0.001). In other words, though all groups acquired fear conditioning, animals that experienced SPS and SPSp learned conditioning faster compared to control rats.

**Figure 2:**
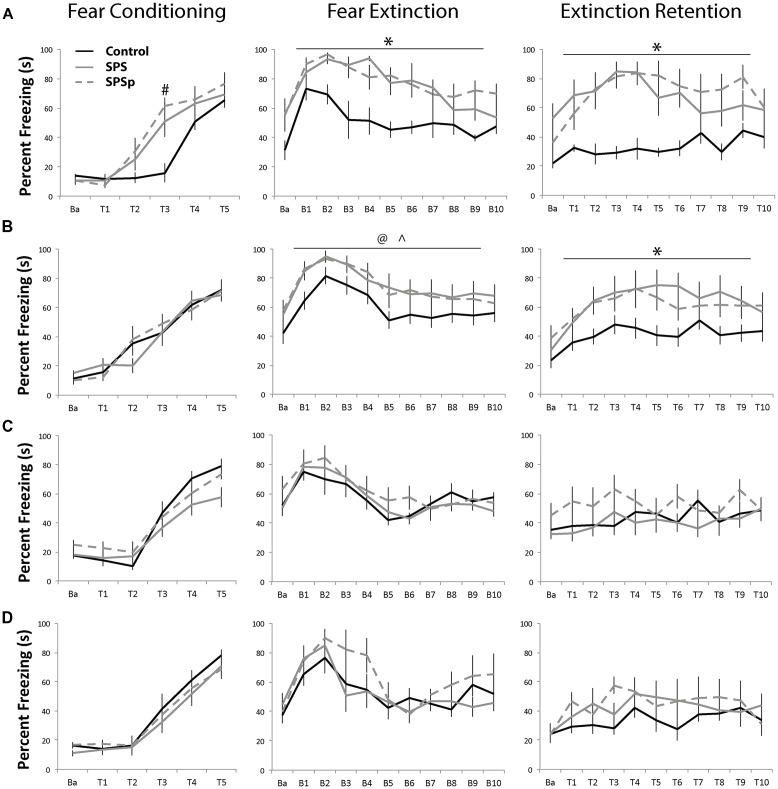
**(A,B)** Adults: all adult rats acquired fear conditioning and learned fear extinction equally. Compared with control rats, rats that experienced single prolonged stress (SPS) and single prolonged stress-predation (SPSp) showed higher freezing, and higher fear trace and slower extinction learning; the experience of the trauma models also resulted in extinction retention deficits. **(C)** Early and **(D)** Mid Adolescents: all adolescent rats learned fear conditioning and extinction, and showed no extinction retention deficits. Ba: baseline; T: trial; B: block of three trials; ^#^indicates different from controls, *p* < 0.05; *indicates different from controls, *p* < 0.05; ^∧^indicates SPSp different from controls, *p* < 0.05; ^@^indicates SPS different from controls, *p* = 0.06.

Fear extinction: all rats showed normal fear learning effects and extinction acquisition (*F* = 9.386, *p* < 0.001). There was a main effect of trauma exposure (*F* = 12.276, *p* < 0.001), such that control rats showed overall lower freezing compared to those that experienced SPS (*p* = 0.001) or SPSp (*p* < 0.001). No difference between the SPS and SPSp groups (*p* = 0.646) and no interaction effects were found (*F* = 0.900, *p* = 0.0587).

Extinction recall: main effects of trial (*F* = 4.789, *p* < 0.001) and trauma exposure (*F* = 9.269, *p* = 0.001) were found; control rats showed overall lower freezing compared to those that experienced SPS (*p* = 0.002) or SPSp (*p* = 0.001). A significant interaction was also found between trial and treatment (*F* = 2.086, *p* = 0.006). While control rats showed constant low freezing levels, those that experienced SPS or SPSp exhibited heightened freezing in response to the initial tones and later within session extinction.

#### Adult Rats, Experiment 2: (Figure 2B)

Fear conditioning: rats showed increasing freezing through consecutive trials (*F* = 61.299, *p* < 0.001). No effects of treatment (*F* = 0.023, *p* = 0.977) or interaction (*F* = 0.984, *p* = 0.461) were found.

Fear extinction: all rats showed recall of fear learning and extinction acquisition (*F* = 14.124, *p* < 0.001). A trend level effect of trauma exposure (*F* = 2.946, *p* = 0.067) was present, indicating that control rats showed overall lower freezing during the extinction session as compared to SPSp animals (*p* = 0.038), and a trend towards lower freezing compared to SPS animals (*p* = 0.064). No interaction effect was found (*F* = 0.418, *p* = 0.999).

Extinction recall: a main effect of trial (*F* = 9.875, *p* < 0.001), and a trend level effect of treatment (*F* = 3.234, *p* = 0.053) was found; control animals froze significantly less than rats that experienced SPS (*p* = 0.040) or SPSp (*p* = 0.037). No interaction effects were found (*F* = 0.927, *p* = 0.552).

#### Mid-Adolescence Rats: (Figure 2C)

Fear conditioning: across the five tone-shock presentations, all animals increased freezing over time (*F* = 76.512, *p* = 0.000). No effect of trauma exposure (*F* = 0.418, *p* = 0.664) or interaction (*F* = 0.301, *p* = 0.907) were found.

Fear extinction: all rats showed normal fear learning and acquisition of extinction (*F* = 10.680, *p* < 0.001). No main effect of trauma exposure (*F* = 1.027, *p* = 0.379) or interactions (*F* = 1.026, *p* = 0.434) were found.

Extinction recall: a main effect of trial was found (*F* = 3.214, *p* = 0.001) that indicated normal extinction retention. No main effect of trauma exposure (*F* = 0.867, *p* = 0.439) or interaction (*F* = 0.946, *p* = 0.530) were found.

#### Early Adolescence Rats: (Figure 2D)

Fear conditioning: a main effect of trial was found (*F* = 78.087, *p* < 0.001); as rats progressed through the tones paired with foot shocks, all acquired conditioning by showing more freezing. No main effect of trauma exposure (*F* = 1.379, *p* = 0.276) or interaction between trauma exposure and trials (*F* = 1.685, *p* = 0.095) on fear learning were found.

Fear extinction: a main effect of trials was found (*F* = 12.215, *p* < 0.001) where animals showed more freezing in the early trials, indicating learned fear, and less freezing in the later trials, indicating acquisition of extinction. No main effect of trauma exposure (*F* = 0.375, *p* = 0.692) or interaction effects (*F* = 0.697, *p* = 0.827) were found.

Extinction recall: no effects of trials (*F* = 1.431, *p* = 0.168), trauma exposure (*F* = 1.351, *p* = 0.281), or interaction (*F* = 1.029, *p* = 0.430) were found.

### Experiment 2: SPS and SPSp Modeling the PTSD Symptom of Enhanced Fear-Potentiated Startle Response (Figure 3)

Acoustic startle: as expected, all rats showed more startle amplitude in the early trials (*F* = 5.651, *p* < 0.001). A main effect (*F* = 6.936, *p* = 0.003) of trauma exposure was found, where control animals showed similar startle amplitude compared to SPSp rats (*p* = 0.699), and overall lower startle amplitude compared to SPS rats (*p* = 0.001). No interaction effect was found (*F* = 1.430, *p* = 0.115).

**Figure 3:**
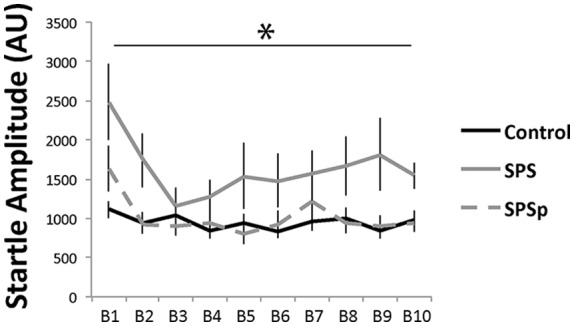
Rats that experienced SPS showed increased startle responses compared to those that experienced SPSp or no trauma; B: block of three acoustic bursts *indicates *p* < 0.05.

## Discussion

Here, we compared the effects of exposure to two trauma models, a well-established PTSD model (SPS) and a novel predation version (SPSp), on fear associated learning in adult, mid adolescent, and early adolescent rodents. Our results demonstrated that a deficit in extinction retention, a hallmark of PTSD seen both in PTSD patients (Milad et al., 2008, 2009) and PTSD rodent models (Knox et al., 2012, 2016; George et al., 2015), was induced by exposure to two types of trauma in adulthood, but not in early or mid adolescence.

Across the three life stages studied, exposure to a novel SPSp paradigm reliably replicated previously documented effects of SPS on fear learning processes (Yamamoto et al., 2009; Knox et al., 2012; George et al., 2015), suggesting that the predation-based trauma model had convergent validity with SPS in this aspect, and that the structural aspects of the SPS such as prolong stressor exposure followed by sensitization period, rather than the specific types of stressors, might be the most pertinent to the development of PTSD-like extinction retention deficits. In addition, our findings show that early and mid adolescent animals were capable of fear conditioning and extinction, as well as of extinction recall, contrasting with previous indications that adolescent rats have impaired extinction learning and extinction retention (McCallum et al., 2010; Baker et al., 2014). When the effects of the two trauma models were compared in adults for a second symptom of PTSD, however, SPS accurately modeled increased fear-potentiated startle (concurrent with prior findings, Khan and Liberzon, 2004), while SPSp did not, suggesting that the effects of trauma type may differ across PTSD symptoms (Butler et al., 1990; Shalev et al., 2000).

There are two potential explanations for deficits in extinction retention after SPS and SPSp in adulthood, but not early or mid adolescents. First, adolescents might be less vulnerable to effects of SPS/SPSp trauma compared with adults. Alternatively, the effects of trauma may manifest differently in adolescence compared with adulthood, or may be delayed and appear only in adulthood (Gluckman et al., 2005; Pattwell et al., 2011). For the first possibility, there is evidence from human literature that support (Green et al., 1991; Liu et al., 2006) and contradict (McFarlane, 1987; Davidson and Smith, 1990) this assertion. If indeed adolescence does not convey increased risk for PTSD, as our data suggests, higher rates of PTSD in adolescents (e.g., Green et al., 1991; Liu et al., 2006), seen in humans, might not reflect common developmental differences, but rather psychological factors, such as poor social support or limited independence to escape adverse conditions, not readily modeled in rodent studies. Supporting this, child abusers are typically in a child’s social network (up to 90%), and risk of recurrent abuse ranges from 9% to 85%, suggesting ongoing adverse conditions may be commonplace after abuse, resulting in distinct effects compared with isolated traumatic incidents (in the United States, Howard, 2000; Hindley et al., 2006; U.S. Department of Health and Human Services, Administration for Children and Families, Administration on Children, Youth and Families, Children’s Bureau, 2017).

Neurobiologically, adolescence can convey differential responsivity to trauma exposure via a number of development-specific mechanisms. In adolescence, the retention of fear extinction information may be less susceptible to trauma because of: (i) protective effects of heightened plasticity, which may facilitate discounting of old information (i.e., fear conditioning) in favor of new information (i.e., fear extinction; Cicchetti, 2016; Panchanathan and Frankenhuis, 2016); or (ii) developmental differences in neural networks recruited for fear extinction and retention. Developmental plasticity is facilitated by BDNF, which peaks in adolescence, and enhances synaptic plasticity, memory formation, and the growth and survival of new neurons (Katoh-Semba et al., 1997; Bekinschtein et al., 2008; Cicchetti, 2016). In adult rats, administration of BNDF to the medial PF (mPFC) can rescue extinction retention deficits (Kabir et al., 2013). This suggests that higher levels of BDNF in adolescence may enhance extinction retention, despite immaturity of the mPFC and hippocampus, attenuating the behavioral effects of trauma. The PFC is very sensitive to stress (Arnsten, 2009; Somerville et al., 2010), but adolescent neural networks have yet to incorporate the role of the PFC, and instead rely on faster developing regions to perform similar cognitive tasks (Spear, 2000, 2013; Tottenham and Galván, 2016). If so, adolescents may be less sensitive to cognitive effects of trauma because they can more effectively compensate for decreases in PFC function, for example during extinction retention. Adolescents also undergo synaptic and receptor density increases, followed by pruning (Cicchetti, 2016). For example, GR binding peaks in the hippocampus during the transition from early to mid adolescence (at 35 days of age in rats; Spear, 2000; Lupien et al., 2009), and adolescent GR levels may be resistant to upward regulation (reviewed in Spear, 2000), a change has been linked to extinction retention deficits in adults (Knox et al., 2012). Thus, adolescents may not exhibit extinction retention deficits following trauma due to lesser GR density changes or GR changes that represent a smaller percentage change compared with adults.

Overall, we found that two different rodent models, an established PTSD model, SPS, and a novel model, SPSp, both replicated adult extinction retention deficits found in PTSD patients (Garfinkel et al., 2014), suggesting that extinction retention deficits might represent PTSD-like phenotype that is not dependent on trauma type. Thus, SPS and SPSp could be valuable models of deficits in extinction retention following trauma in adulthood. Yet, only SPS increased acoustic startle response, suggesting that trauma type may have differential effects on mechanisms underlying distinct PTSD symptoms. To fully ascertain the utility of the SPSp model, future investigations will need to assess further behavioral and physiological symptoms of PTSD following SPSp exposure. Despite the novelty of the SPSp model, following SPSp and the established SPS model, adults exhibited PTSD-like extinction retention deficits, whereas adolescent rats showed no such deficits following either trauma type, suggesting that adolescents may be resilient to cognitive effects of these types of trauma exposure.

## Definitions

*Extinction*—the ability to extinguish fear responses when presented with a cue that was previously negatively reinforced, but is no longer associated with a negative stimulus.

*Extinction retention*—the ability to recall and apply extinction to attenuate fear responses upon re-exposure to the extinction context.

## Data Availability

The raw data supporting the conclusions to the present manuscript will be provided to any qualified researcher upon request.

## Author Contributions

All authors contributed to the design of the study. SN, CC and LC conducted the study. CC analyzed the data. CC, LC and IL wrote the manuscript.

## Conflict of Interest Statement

The authors declare that the research was conducted in the absence of any commercial or financial relationships that could be construed as a potential conflict of interest.

## Abbreviations

BDNF: brain-derived neurotrophic factor
GR: glucocorticoid receptor
PFC: prefrontal cortex
PTSD: posttraumatic stress disorder
SPS: single prolonged stress
SPSp: single prolonged stress-predation.
